# Changes in American Society of Anesthesiologists Physical Status Distribution and Prognostic Performance During the 2024 South Korean Healthcare Crisis: A Large-Scale Retrospective Cohort Study

**DOI:** 10.3390/jcm15114261

**Published:** 2026-05-31

**Authors:** Chan-Sik Kim, Sang-Wook Lee

**Affiliations:** Department of Anesthesiology and Pain Medicine, Asan Medical Center, University of Ulsan College of Medicine, 88 Olympic-ro 43-gil, Songpa-gu, Seoul 05505, Republic of Korea; chansik.kim@amc.seoul.kr

**Keywords:** American Society of Anesthesiologists Physical Status, risk assessment, postoperative mortality, propensity score, healthcare crisis

## Abstract

**Background**: The American Society of Anesthesiologists Physical Status (ASA-PS) classification is widely used for perioperative risk stratification but is subject to inter-rater variability. The 2024 South Korean medical crisis abruptly shifted preoperative ASA-PS assessment from resident-led to specialist-centered care, providing a natural opportunity to examine how this transition affected ASA-PS distribution and prognostic performance. **Methods**: In this single-center retrospective cohort study, surgical patients during the pre-crisis (January 2022–December 2023) and crisis (March 2024–August 2025) periods were matched 1:2 by propensity score on age, sex, Charlson Comorbidity Index, surgical specialty, emergency status, and anesthesia type. The primary outcome was 30-day mortality; secondary outcomes were postoperative intensive care unit (ICU) admission and length of stay. ASA-PS discrimination was compared between periods using DeLong’s test, and ASA × crisis interaction terms were assessed by the likelihood ratio test. **Results**: A total of 53,895 cases (35,930 pre-crisis; 17,965 crisis) were matched, with all post-matching standardized mean differences below 0.1. ASA-PS demonstrated higher discrimination for 30-day mortality during the crisis than the pre-crisis period (area under the curve [AUC], 0.891 [0.863–0.919] vs. 0.827 [0.803–0.851]; ΔAUC = 0.064, *p* < 0.001). The ASA-PS × crisis interaction remained significant after adjustment (*p* = 0.014). Discrimination for ICU admission was similar between periods. **Conclusions**: ASA-PS classifications assigned during the crisis period were associated with higher discrimination for 30-day mortality than those from the pre-crisis period, suggesting that the operational performance of perioperative risk-assessment tools may vary with evaluator context and broader healthcare system conditions.

## 1. Introduction

The American Society of Anesthesiologists Physical Status (ASA-PS) classification has served as a cornerstone of perioperative risk assessment for more than 80 years. First proposed by Saklad in 1941 [1] and subsequently formalized into its current six-tier structure by Dripps et al. in 1963 [2], the ASA-PS provides a standardized clinical framework for communicating a patient’s preoperative physiological status [3]. Extensive literature has established the classification as an independent predictor of postoperative morbidity and mortality [4,5], with a meta-analysis reporting a pooled area under the receiver operating characteristic curve (AUC) of 0.736 for predicting postoperative mortality [6]. Despite its widespread use, the system has been criticized for its inherent subjectivity, which can lead to substantial inter-rater variability [7], and recent work suggests that the clinical experience of the evaluator may influence ASA-PS assignment [3,8].

The South Korean healthcare system experienced an unprecedented disruption in 2024, a situation rarely observed in modern medical systems [9]. In February 2024, a conflict over the government’s proposal to expand medical school enrollment quotas resulted in the collective resignation of more than 90% of medical residents, who had previously constituted 30–40% of the physician workforce in tertiary hospitals. This abrupt exodus exposed structural vulnerabilities within the national healthcare system and triggered substantial changes across the medical landscape [9]. Recent analyses have reported significant reductions in medical research output [10] and surgical case volumes during this period [11]. The absence of residents forced an abrupt transition to a specialist-centered care model, in which attending physicians and intensivists assumed markedly increased clinical workloads and reorganized critical care delivery processes [12].

Although prior studies of the 2024 medical crisis have focused mainly on reductions in emergency services, surgical volume, and academic productivity [10,11,13,14], the potential effects of these workforce changes on the accuracy of perioperative clinical assessment remain largely unexplored. Capitalizing on this unique natural experiment, we examined whether the abrupt transition in clinical workforce composition was associated with changes in ASA-PS distribution and its prognostic performance for postoperative outcomes—specifically 30-day mortality and intensive care unit (ICU) admission—in a large surgical cohort at a tertiary referral center. Given the concurrent system-level changes during this period, causal attribution to any single factor requires caution; rather, our aim was to describe how a healthcare workforce transition may be associated with changes in the distribution and prognostic performance of preoperative risk-stratification tools.

## 2. Methods

### 2.1. Ethics Statement and Study Design

This study was approved by the Institutional Review Board of Asan Medical Center (AMC) (IRB No. 2026-0147). The requirement for informed consent was waived owing to the retrospective nature of the study. All procedures were conducted in accordance with the principles of the Declaration of Helsinki and institutional data security policies. This single-center retrospective cohort study analyzed surgical patients treated at AMC, a large tertiary academic referral hospital. The study is reported in accordance with the Strengthening the Reporting of Observational Studies in Epidemiology (STROBE) statement [15].

### 2.2. Study Population and Eligibility Criteria

The study population was categorized into two periods according to the predominant evaluator of the ASA-PS classification: the pre-crisis period (1 January 2022 to 31 December 2023), during which assessments were predominantly performed by anesthesiology residents under specialist supervision, and the medical crisis period (1 March 2024 to 31 August 2025), during which assessments were primarily performed by attending specialists following the workforce transition. All patients who underwent surgery requiring anesthesia during the study periods were screened for eligibility. Records with missing or ambiguous ASA-PS classifications that precluded identification of the primary evaluator were excluded. Patients classified as ASA V or VI were also excluded to minimize bias in prognostic performance comparisons, as these categories represent extreme physiological conditions or terminal states.

### 2.3. Data Collection and Definition of Variables

Data were extracted from the institutional electronic medical record structured database through the AMC Clinical Data Warehouse, including surgical and anesthetic records, diagnoses, mortality data, and ICU admission records. The primary explanatory variable was the ASA-PS classification documented in the anesthetic record. Baseline covariates included age, sex, surgical specialty, emergency surgery status, and anesthesia category. Comorbidity burden was quantified using the Charlson Comorbidity Index (CCI), calculated from International Classification of Diseases, 10th Revision (ICD-10) codes recorded prior to the index surgery date [16]. For propensity score estimation, CCI was treated as a categorical variable (0, 1, 2, or ≥3) to account for the non-linear relationship between comorbidity burden and group assignment. Anesthesia type was categorized as general anesthesia, regional anesthesia, or monitored anesthesia care.

### 2.4. Study Outcomes

The primary outcome was 30-day mortality, defined as all-cause death within 30 days after surgery. Mortality data were obtained from the institutional electronic medical records and cross-referenced with national death registry data through the Clinical Data Warehouse to ensure completeness of follow-up beyond in-hospital events. Secondary outcomes were postoperative ICU admission and ICU length of stay (LOS). ICU LOS was reported as median (interquartile range [IQR]) given its right-skewed distribution.

### 2.5. Statistical Analysis

To reduce baseline imbalance and potential confounding between the pre-crisis and crisis periods, propensity score matching (PSM) was performed using a logistic regression model. Covariates included age, sex, CCI (categorical), surgical specialty (top 10 departments by volume, with the remainder grouped as “other”), emergency surgery status, and anesthesia category. Categorical covariates were entered as dummy variables using one-hot encoding to avoid imposing artificial ordinal relationships among nominal categories [17]. A 1:2 nearest-neighbor greedy matching algorithm without replacement was applied on the logit of the propensity score, with a caliper of 0.2 standard deviations of the logit [17]. Common support was ensured by restricting matching to the overlapping range of propensity scores between the two groups; crisis-period patients without two suitable matches within the caliper were excluded from the matched cohort.

Covariate balance before and after matching was assessed using standardized mean differences (SMDs); an absolute SMD > 0.1 was considered indicative of meaningful imbalance [17]. After matching, perioperative characteristics and postoperative outcomes were compared between groups, and ASA-PS distributions before and after matching were compared using χ^2^ tests.

To evaluate the prognostic performance of ASA-PS for postoperative outcomes, receiver operating characteristic (ROC) curve analyses were performed separately for each period, and the AUC with 95% confidence intervals was calculated using DeLong’s method [18]. To formally compare ASA-PS discrimination between the two periods, DeLong’s test for two independent ROC curves was applied, providing the difference in AUC (ΔAUC), its standard error, z-statistic, and two-sided *p* value. Given the fixed sample, we conducted a sensitivity analysis estimating the minimum detectable difference in AUC rather than a circular post hoc power calculation; with the observed event counts, the study had ≥80% power to detect an AUC difference of approximately 0.05 by DeLong’s test (two-sided α = 0.05). ASA class-specific mortality and ICU admission rates were calculated for each period in both eligible and matched cohorts.

To assess whether the association between ASA-PS and postoperative outcomes differed by crisis status, logistic regression models including an ASA × crisis interaction term were constructed. Both unadjusted (ASA-PS × crisis status) and adjusted (additionally controlling for age, sex, CCI, and emergency surgery status) models were evaluated. For each outcome, a reduced model was compared with a full model that additionally included the ASA-PS × crisis interaction; likelihood ratio tests were used to evaluate significance. These analyses were performed in both the eligible and the propensity score-matched cohorts. All analyses were performed using Python (version 3.10.16) within a secure institutional research environment, and a two-sided *p* < 0.05 was considered statistically significant.

## 3. Results

### 3.1. Study Population and Propensity Score Matching

**Figure 1:**
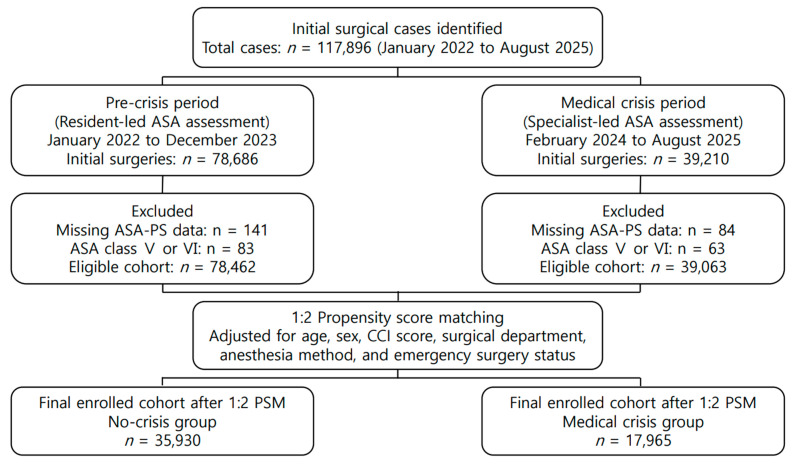
Flow diagram of study population selection and propensity score matching. ASA-PS—American Society of Anesthesiologists physical status classification; CCI—Charlson comorbidity index; PSM—propensity score matching.

The study population selection process is illustrated in Figure 1. A total of 117,896 surgical cases were initially identified from the institutional surgical database covering January 2022 to August 2025. These were categorized into a pre-crisis cohort (resident-led, *n* = 78,686) and a crisis cohort (specialist-led, *n* = 39,210). After applying exclusion criteria (missing ASA-PS, *n* = 225; ASA V/VI, *n* = 146), 117,525 cases remained eligible for analysis (78,462 pre-crisis and 39,063 crisis).

Propensity scores were estimated using a logistic regression model incorporating 19 variables (age, sex, emergency surgery status, three CCI dummy variables, ten surgical-department dummy variables, and three anesthesia-type dummy variables); a caliper of 0.049 (0.2 × SD of the logit propensity score = 0.2 × 0.244) was applied. Of 39,063 crisis-period patients, 17,965 (46.0%) were successfully matched to 35,930 pre-crisis controls, yielding a final matched cohort of 53,895 surgical cases (Table 1). The 21,098 unmatched crisis-period patients (54.0%) had markedly lower comorbidity and acuity than the matched patients (CCI 0.2 ± 1.0 vs. 1.2 ± 2.3; emergency surgery 4.0% vs. 14.1%; regional anesthesia 10.2% vs. 2.7%; Table 1), reflecting preferential exclusion of low-risk elective procedures that lacked comparable pre-crisis controls within the caliper.

Before matching, most baseline covariates were reasonably balanced (all SMD < 0.2), with the exception of anesthesia type (general anesthesia SMD = 0.120; regional anesthesia SMD = 0.162). After matching, all baseline covariates achieved acceptable balance (all SMD < 0.1; Table 1). The covariate balance improvement is visualized in Supplementary Figure S1, and propensity score overlap before and after matching is shown in Supplementary Figure S2.

### 3.2. Perioperative Characteristics and Clinical Outcomes

**Figure 2:**
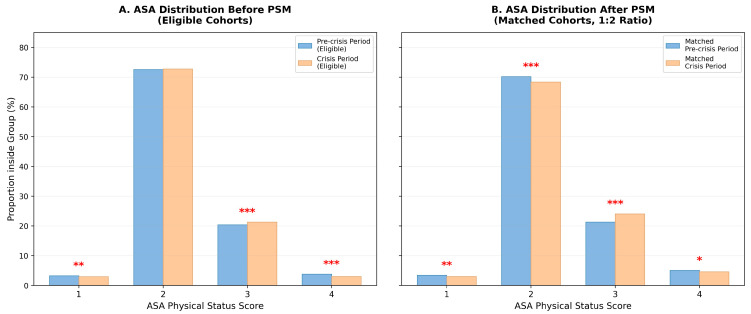
Distribution of ASA physical status scores in the pre-crisis and crisis periods before and after 1:2 propensity score matching. Panel (**A**) shows the within-group proportional distribution of ASA classes in the eligible cohorts before matching, and Panel (**B**) shows the within-group proportional distribution in the matched cohorts. Asterisks indicate statistically significant between-group differences for each ASA category (* *p* < 0.05, ** *p* < 0.01, *** *p* < 0.005). ASA, American Society of Anesthesiologists; PSM, propensity score matching.

After PSM, key preoperative characteristics were substantially balanced between groups (Table 1). The matched pre-crisis and crisis cohorts had similar CCI scores (1.1 ± 2.2 vs. 1.2 ± 2.3, SMD = 0.012). Differences in ASA-PS distribution remained between the two periods even after matching (Figure 2): the matched crisis cohort contained a modestly higher proportion of ASA III patients (24.1% vs. 21.3%, SMD = 0.066) and a lower proportion of ASA II patients (68.3% vs. 70.2%, SMD = 0.041); none of these category-specific differences exceeded the 0.1 SMD threshold.

Crude postoperative outcomes in the matched cohort were as follows: 30-day mortality was lower during the crisis than the pre-crisis period (0.6% [111/17,965] vs. 0.8% [299/35,930]). Unplanned postoperative ICU admission was higher during the crisis (18.5% vs. 14.7%). Among ICU-admitted patients, median ICU LOS was 1.0 (IQR, 1.0–2.0) days in the pre-crisis group and 2.0 (IQR, 2.0–4.0) days in the crisis group.

### 3.3. ASA Class-Specific Outcome Rates

ASA class-specific 30-day mortality and ICU admission rates in the matched cohort are presented in Table 2. In both periods, 30-day mortality increased progressively with ASA class, and no deaths were observed among ASA I patients. Notably, mortality at ASA II was 0.06% (crisis) vs. 0.21% (pre-crisis), whereas mortality at ASA IV was similar between periods (7.84% vs. 8.00%). This pattern suggests that the higher AUC during the crisis period reflects better discrimination at the lower end of the risk spectrum rather than improved identification of high-risk patients (Supplementary Table S2 presents the corresponding data for the eligible cohort).

### 3.4. Discriminatory Performance of ASA-PS

**Figure 3:**
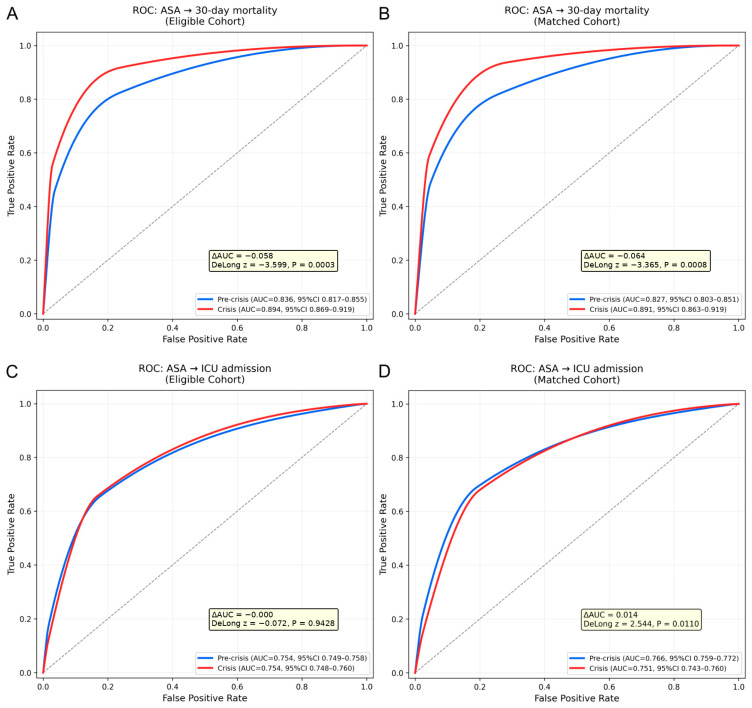
Receiver operating characteristic curves of ASA physical status for predicting 30–day mortality and ICU admission before and after propensity score matching. Panels (**A**,**B**) show ROC curves for 30–day mortality in the eligible and matched cohorts, respectively. Panels (**C**,**D**) show ROC curves for ICU admission in the eligible and matched cohorts, respectively. AUC values are presented with 95% confidence intervals (DeLong method). DeLong’s test *p*–values for between-period AUC comparison are displayed on each panel. The diagonal dashed line indicates the line of no discrimination (AUC = 0.5). ASA—American Society of Anesthesiologists; ICU—intensive care unit; ROC—receiver operating characteristic; AUC—area under the ROC curve.

The discriminatory performance of ASA-PS for predicting 30-day mortality and ICU admission is summarized in Table 3 and Figure 3. In the eligible cohort, ASA-PS demonstrated higher discrimination for 30-day mortality during the crisis than the pre-crisis period (AUC, 0.894 [95% CI, 0.869–0.919] vs. 0.836 [0.817–0.855]; ΔAUC = 0.058; z = −3.60; DeLong *p* = 0.0003). In the matched cohort, the difference was preserved (AUC, 0.891 [0.863–0.919] vs. 0.827 [0.803–0.851]; ΔAUC = 0.064; z = −3.37; *p* = 0.0008). The crisis-period AUC was closely consistent between the eligible (0.894) and matched (0.891) cohorts, supporting the robustness of this finding.

For ICU admission, AUC values in the eligible cohort were nearly identical between periods (0.754 [0.749–0.758] vs. 0.754 [0.748–0.760]; DeLong *p* = 0.943). In the matched cohort, ASA-PS demonstrated modestly higher discrimination for ICU admission in the pre-crisis cohort (0.766 [0.759–0.772]) than in the crisis cohort (0.751 [0.743–0.760]; ΔAUC = 0.014; *p* = 0.011). Overall, the prognostic performance of ASA-PS for 30-day mortality was stronger during the medical crisis period, whereas its predictive performance for ICU admission was broadly similar between periods and showed only a modest reversal in the matched cohort.

### 3.5. Interaction Analysis

To evaluate whether the association between ASA-PS and postoperative outcomes differed according to crisis status, likelihood ratio tests were conducted by comparing logistic regression models with and without an ASA × crisis interaction term (Supplementary Table S1). For 30-day mortality, the interaction term was statistically significant in the eligible cohort in both unadjusted (χ^2^ = 16.20, *p* < 0.001) and adjusted (χ^2^ = 7.33, *p* = 0.007) models, indicating that the association between ASA class and postoperative mortality differed significantly by crisis status even after controlling for age, sex, CCI, and emergency surgery status. In the matched cohort, the interaction remained significant in both unadjusted (χ^2^ = 10.38, *p* = 0.001) and adjusted (χ^2^ = 6.09, *p* = 0.014) models. For ICU admission, the interaction was statistically significant only in the unadjusted model of the eligible cohort (χ^2^ = 5.69, *p* = 0.017), and not in the adjusted eligible cohort (*p* = 0.090) or in either matched-cohort model (unadjusted *p* = 0.157; adjusted *p* = 0.140), suggesting that the differential association between ASA class and ICU admission attenuated after covariate adjustment and matching.

## 4. Discussion

In this single-center retrospective cohort study of more than 117,000 surgical cases, we examined whether the distribution and prognostic performance of ASA-PS classification differed according to medical crisis status, which also corresponded to a shift in the predominant evaluator from a resident-led to a specialist-led model. Two main findings emerged. First, the distribution of ASA classes differed between the pre-crisis and crisis periods even after propensity score matching achieved adequate covariate balance, suggesting that ASA assignment patterns were not identical across the two clinical environments. Second, ASA classification during the crisis period was associated with higher discrimination for 30-day mortality than during the pre-crisis period (ΔAUC = 0.064, *p* < 0.001), whereas differences in ICU admission prediction were smaller and less consistent. Importantly, the higher AUC should not be interpreted as indicating that specialists simply assigned uniformly higher ASA classes; rather, ASA assignments during the crisis period were more closely associated with observed mortality risk, particularly at the lower end of the risk spectrum.

These findings should be interpreted within the context of the extraordinary medical crisis that began in South Korea in 2024. Following the government’s announcement of a planned expansion of 2000 medical school admissions annually, approximately 9000 residents and fellows resigned in February 2024, with more than 9600 still resigned as of late May 2024. This unprecedented workforce disruption led to substantial nationwide challenges in hospital staffing and care delivery [9]. Contemporary analyses have characterized the situation as a systemic crisis in the Korean healthcare system rather than a conventional labor dispute, and have documented disruptions to critical care delivery, including reductions in ICU staffing, increased intensivist working hours and night-duty burdens, and expanded clinical responsibilities of nurse practitioners [12]. Concurrent declines in domestic medical research output [10,19,20] and disruption of surgical residency training and oncologic care delivery [13,14] have also been described. Collectively, these reports indicate that the 2024 crisis represented a broad systemic disruption with potential implications not only for healthcare delivery but also for clinical assessment practices, supervisory structures, and decision-making processes.

ASA-PS classification remains one of the most widely used perioperative risk-assessment tools in anesthesiology. Although the American Society of Anesthesiologists notes that ASA-PS alone does not fully predict perioperative risk, it remains a practical and broadly accepted summary measure of baseline illness severity. Prior studies have consistently shown that increasing ASA class is associated with higher postoperative mortality [4,5], and meta-analytic evidence supports its utility as a predictor of postoperative death [6,21]. However, ASA classification is also known to be subject to inter-rater variability [3,7]. In a survey-based national study by De Cassai et al., ASA assignments varied according to anesthesiologist experience, with overall inter-rater reliability being only weak to moderate [8]. That study, however, relied on hypothetical clinical scenarios rather than real-world perioperative practice and could therefore not determine whether evaluator-dependent differences in ASA assignment translate into differences in prognostic performance in actual patients. In contrast, our analysis evaluated whether ASA assignments in routine clinical care across more than 117,000 surgical cases differed in their association with actual postoperative outcomes during a natural shift in evaluator structure. From this perspective, our findings provide clinically grounded evidence that evaluator context may be associated with differences not only in the distribution of ASA classifications but also in their real-world prognostic meaning.

From a clinical and quality-of-care perspective, the magnitude of the AUC difference observed here is non-trivial. A ΔAUC of 0.064 in the matched cohort represents a meaningful upward shift in discrimination for postoperative mortality, comparable to the gain achieved by adding several objective laboratory variables to ASA-PS in published risk-prediction models [22,23]. The ASA class-specific mortality data (Table 3) provide additional context: in the matched cohort, mortality at ASA II was 0.06% (crisis) versus 0.21% (pre-crisis), whereas rates at ASA IV were similar between periods (7.84% vs. 8.00%). This pattern indicates that the higher AUC during the crisis period was attributable to better discrimination at the lower end of the risk spectrum rather than to differences in identifying high-risk patients. Although mortality differences at ASA II are small in absolute terms, they translate into clinically meaningful relative differences in low-risk surgical populations, which represent the majority of surgical case volume at tertiary hospitals.

The observation that ICU admission discrimination did not improve in parallel with mortality discrimination merits further consideration. ICU admission decisions reflect both patient physiology and a wide array of system-level factors—including bed availability, surgeon and intensivist preferences, and institutional triage patterns—many of which were themselves disrupted during the medical crisis [12]. Consequently, ICU admission may be a less faithful indicator of intrinsic patient risk than 30-day mortality and a noisier outcome for assessing the prognostic performance of any preoperative tool in this setting. The differential pattern observed for mortality versus ICU admission therefore suggests that the change in mortality discrimination reflects genuine patient-level prognostic information rather than a nonspecific artifact of crisis-related disruption of ICU triage.

The broader significance of this study is that the Korean medical crisis created an unusually large-scale natural experiment in clinical workforce composition. Few healthcare systems experience such a sudden and nationwide transition in frontline evaluator structure. Because ASA-PS is among the most routinely used perioperative assessment tools globally, examining how its distribution and prognostic performance changed under these conditions offers a unique opportunity to understand how workforce disruption may influence core clinical judgment. Our findings are consistent with the hypothesis that evaluator context can influence how ASA-PS functions as a prognostic variable in clinical research, quality assessment, and perioperative risk adjustment—with implications for studies that pool ASA-PS data across institutions, time periods, or healthcare systems with differing evaluator structures.

At the same time, whether this improvement in discrimination reflects evaluator experience or other concurrent changes cannot be determined from our data. The shift from resident-led to specialist-led evaluation occurred alongside reduced surgical volume and altered workflow, and lower case volume alone may have permitted more thorough preoperative assessment irrespective of the evaluator’s seniority. The features noted above—improvement concentrated among lower-risk patients, specific to mortality rather than ICU admission, and persisting after matching on measured case-mix—are compatible with a contribution from evaluator experience but do not establish it as the dominant cause. Disentangling evaluator-level from system-level effects will require studies designed to vary evaluator experience independently of system conditions.

Several limitations should be acknowledged. Firstly, this study was conducted at a single center; although AMC is a large tertiary referral hospital with a high surgical volume and the matched cohort included nearly 54,000 cases, the findings may not generalize to community hospitals or to healthcare systems with different evaluator structures, and external replication is needed. Nonetheless, AMC is one of the highest-volume surgical centers in the Republic of Korea, and the size and granularity of the cohort strengthen the internal reliability of the observed patterns. Secondly, although PSM achieved adequate covariate balance, residual confounding from unmeasured variables is likely to remain. The medical crisis affected numerous aspects of clinical care—including staff workload, fatigue, supervision patterns, workflow disruption, and institutional adaptation—that were not fully captured in our dataset, such as within-ASA-class disease severity and the selection of which patients proceeded to surgery during the crisis. Moreover, because the comparison spans two distinct time periods, secular trends unrelated to the evaluator transition cannot be excluded (temporal confounding). Accordingly, the observed differences in prognostic performance should not be attributed solely to evaluator experience or specialty status. Thirdly, the crisis period variable functions as a composite surrogate for multiple simultaneous changes (evaluator transition, case-mix shift, workload intensification, and institutional adaptation), and the observed association cannot be causally attributed to evaluator expertise alone. Fourthly, our dataset did not include a variable reflecting surgical complexity (such as the relative value unit), which would have improved matching and further reduced residual confounding. Fifthly, ASA-PS is inherently subjective and may reflect contextual factors beyond patient condition, including institutional culture, local practice patterns, and documentation behavior. Finally, the matching rate of 46% indicates that the matched cohort represents a selected subset of crisis-period patients, and unmeasured differences between matched and unmatched patients may limit generalizability. As shown in Table 1, unmatched crisis-period patients were substantially lower-acuity than matched patients, so the matched cohort over-represents higher-acuity surgery and is not representative of the entire crisis-period surgical population. Reassuringly, ASA-PS discrimination for 30-day mortality in the full eligible cohort (AUC 0.894) was nearly identical to that in the matched cohort (AUC 0.891), indicating that the principal finding was not driven by this matching-based selection.

## 5. Conclusions

In this large single-center retrospective cohort study, ASA-PS classifications assigned during the 2024 medical crisis period were associated with higher discrimination for 30-day postoperative mortality than ASA-PS classifications assigned during the pre-crisis period, a finding that was robust across eligible and matched cohorts and persisted after covariate adjustment. Although these results should be interpreted cautiously given the observational design and multiple concurrent system-level changes, they provide a real-world example of how abrupt changes in the clinical workforce may influence not only care delivery but also the operational performance and prognostic meaning of a widely used risk-classification system. Future multicenter studies incorporating quasi-experimental methods and additional surgical risk variables are needed to determine whether these findings are reproducible and to further disentangle evaluator-related effects from broader system-level consequences of the medical crisis.

## Figures and Tables

**Table 1:** Baseline and perioperative characteristics, and postoperative outcomes of surgical patients across the pre-crisis and medical crisis periods before and after 1:2 propensity score matching, and characteristics of unmatched crisis-period patients.

Variables	Pre-Crisis (Eligible)	Crisis (Eligible)	SMD	Pre-Crisis (Matched)	Crisis (Matched)	SMD	Crisis (Unmatched)	SMD (M vs. U)
*N*	78,462	39,063		35,930	17,965		21,098	
Age (years)	57.1 ± 15.0	55.9 ± 15.6	0.080	56.3 ± 16.5	56.4 ± 16.5	0.008	55.5 ± 14.8	0.070
Sex (female), *n* (%)	42,162 (53.7)	22,290 (57.1)	0.067	19,082 (53.1)	9576 (53.3)	0.004	12,714 (60.3)	0.142
Anesthesia methods, *n* (%)								
General	74,345 (94.8)	35,844 (91.8)	0.120	34,033 (94.7)	16,988 (94.6)	0.007	18,856 (89.4)	0.180
Regional	2547 (3.2)	2646 (6.8)	0.162	930 (2.6)	485 (2.7)	0.007	2161 (10.2)	0.309
MAC	1570 (2.0)	573 (1.5)	0.041	967 (2.7)	492 (2.7)	0.003	81 (0.4)	0.214
CCI	0.7 ± 1.8	0.6 ± 1.8	0.025	1.1 ± 2.2	1.2 ± 2.3	0.012	0.2 ± 1.0	0.560
ASA physical status, *n* (%)								
ASA I	2542 (3.2)	1147 (2.9)	0.018	1243 (3.5)	542 (3.0)	0.025	605 (2.9)	0.000
ASA II	56,947 (72.6)	28,411 (72.7)	0.003	25,221 (70.2)	12,272 (68.3)	0.041	16,139 (76.5)	0.187
ASA III	15,981 (20.4)	8318 (21.3)	0.023	7653 (21.3)	4322 (24.1)	0.066	3996 (18.9)	0.128
ASA IV	2992 (3.8)	1187 (3.0)	0.043	1813 (5.0)	829 (4.6)	0.020	358 (1.7)	0.175
Emergency, *n* (%)	6945 (8.9)	3374 (8.6)	0.008	4699 (13.1)	2526 (14.1)	0.029	848 (4.0)	0.356
Postoperative outcomes								
30-day mortality	462 (0.6)	159 (0.4)	0.026	299 (0.8)	111 (0.6)	0.025	48 (0.2)	0.051
ICU admission	10,723 (13.7)	6106 (15.6)	0.056	5277 (14.7)	3324 (18.5)	0.103	2782 (13.2)	0.153
ICU LOS, median (IQR)	1.0 (1.0–2.0)	2.0 (2.0–3.0)		1.0 (1.0–2.0)	2.0 (2.0–4.0)		2.0 (2.0–3.0)	

Data represent mean ± standard deviation, median (IQR), or number (percentage). SMD balance assessment pertains to baseline covariates used for matching. SMD—standardized mean difference; CCI—Charlson comorbidity index; ASA—American Society of Anesthesiologists physical status; ICU—intensive care unit; LOS—length of stay; IQR—interquartile range; MAC—monitored anesthesia care. All post-matching SMDs for baseline covariates were below the 0.1 threshold for meaningful imbalance. The rightmost SMD column compares matched versus unmatched crisis-period patients and reflects the selection imposed by caliper matching, not covariate balance between periods.

**Table 2:** ASA class-specific 30-day mortality and ICU admission rates in the propensity score-matched cohort.

ASA Class	Pre-Crisis			Crisis		
	*N*	30-Day Mortality, *n* (%)	ICU Admission, *n* (%)	N	30-Day Mortality, *n* (%)	ICU Admission, *n* (%)
I	1243	0 (0.0)	27 (2.2)	542	0 (0.0)	8 (1.5)
II	25,221	54 (0.21)	1614 (6.4)	12,272	7 (0.06)	1063 (8.7)
III	7653	100 (1.31)	2527 (33.0)	4322	39 (0.90)	1766 (40.9)
IV	1813	145 (8.00)	1109 (61.2)	829	65 (7.84)	487 (58.7)
Total	35,930	299 (0.83)	5277 (14.7)	17,965	111 (0.62)	3324 (18.5)

Values represent *n* (%). ASA—American Society of Anesthesiologists physical status; ICU—intensive care unit.

**Table 3:** Discriminatory performance of ASA-PS for predicting postoperative outcomes: AUC comparison with DeLong’s test.

Cohort	Outcome	Pre-Crisis AUC (95% CI)	Crisis AUC (95% CI)	ΔAUC (z-Statistic)	*p* Value
Eligible	30-day mortality	0.836 (0.817–0.855)	0.894 (0.869–0.919)	0.058 (z = −3.60)	0.0003
	ICU admission	0.754 (0.749–0.758)	0.754 (0.748–0.760)	0.000 (z = −0.07)	0.943
Matched	30-day mortality	0.827 (0.803–0.851)	0.891 (0.863–0.919)	0.064 (z = −3.37)	0.0008
	ICU admission	0.766 (0.759–0.772)	0.751 (0.743–0.760)	−0.014 (z = +2.54)	0.011

ΔAUC, difference in AUC, was calculated as AUC in the crisis period—AUC in the pre-crisis period. AUC—area under the receiver operating characteristic curve; CI—confidence interval; ΔAUC—difference in AUC (pre-crisis minus crisis). A negative ΔAUC indicates higher AUC in the crisis period. DeLong’s test was used for formal comparison between two independent groups.

## Data Availability

The dataset used in this study is not publicly available because of restrictions imposed by the Institutional Review Board of Asan Medical Center under Korean ethical guidelines. Reasonable requests for data access may be directed to the corresponding author and will be considered in accordance with applicable institutional and regulatory policies.

## Supplementary Materials

The following supporting information can be downloaded at https://www.mdpi.com/article/10.3390/jcm15114261/s1: Supplementary Table S1: Likelihood ratio test for interaction between ASA physical status and medical crisis status in predicting postoperative outcomes. Supplementary Table S2: ASA class-specific 30-day mortality and ICU admission rates in the eligible cohort before propensity score matching. Supplementary Figure S1: Standardized mean differences (Love plot) for all covariates before and after propensity score matching. Supplementary Figure S2: Propensity score distributions before and after matching for the crisis and pre-crisis groups.
