# Supplementary material for: Changes in American Society of Anesthesiologists Physical Status Distribution and Prognostic Performance During the 2024 South Korean Healthcare Crisis: A Large-Scale Retrospective Cohort Study
Source: J Clin Med. 2026 May 31;15(11):4261. doi: 10.3390/jcm15114261 (PMC13258368; doi:10.3390/jcm15114261)

Supplementary Materials

Changes in American Society of Anesthesiologists Physical Status Distribution and Prognostic Performance During the 2024 South Korean Healthcare Crisis: A Large-Scale Retrospective Cohort Study

Chan-Sik Kim and Sang-Wook Lee

Supplementary Table S1. Likelihood ratio test for interaction between ASA physical status and medical crisis status in predicting postoperative outcomes.

Cohort	Outcome	Test	χ^2	df	P for interaction
Eligible	30-day mortality	Unadjusted LRT	16.197	1	<0.001
		Adjusted LRT	7.331	1	0.007
	ICU admission	Unadjusted LRT	5.688	1	0.017
		Adjusted LRT	2.870	1	0.090
Matched	30-day mortality	Unadjusted LRT	10.375	1	0.001
		Adjusted LRT	6.093	1	0.014
	ICU admission	Unadjusted LRT	2.002	1	0.157
		Adjusted LRT	2.179	1	0.140

Reduced model: outcome ~ ASA + medical crisis status (± covariates).

Full model: additionally includes ASA × medical crisis status interaction.

Adjusted models additionally control for age, sex, CCI, and emergency surgery status.

ASA-PS, American Society of Anesthesiologists physical status; LRT, likelihood ratio test; ICU, intensive care unit.

Supplementary Table S2. ASA class-specific 30-day mortality and ICU admission rates in the eligible cohort before propensity score matching.

ASA class	Pre-crisis			Crisis		
	N	30-day mortality, n (%)	ICU admission, n (%)	N	30-day mortality, n (%)	ICU admission, n (%)
I	2,542	0 (0.00)	55 (2.2)	1,147	0 (0.00)	15 (1.3)
II	56,947	81 (0.14)	3,642 (6.4)	28,411	13 (0.05)	2,088 (7.3)
III	15,981	169 (1.06)	5,163 (32.3)	8,318	58 (0.70)	3,309 (39.8)
IV	2,992	212 (7.09)	1,863 (62.3)	1,187	88 (7.41)	694 (58.5)
**Total**	**78,462**	**462 (0.59)**	**10,723 (13.7)**	**39,063**	**159 (0.41)**	**6,106 (15.6)**

Values represent n (%). ASA, American Society of Anesthesiologists physical status; ICU, intensive care unit.

Supplementary Figure S1. Standardized mean differences (Love plot) for all covariates before and after propensity score matching. Open red circles indicate SMD before matching; filled blue circles indicate SMD after matching. The dashed red line indicates the SMD = 0.1 threshold for acceptable balance.

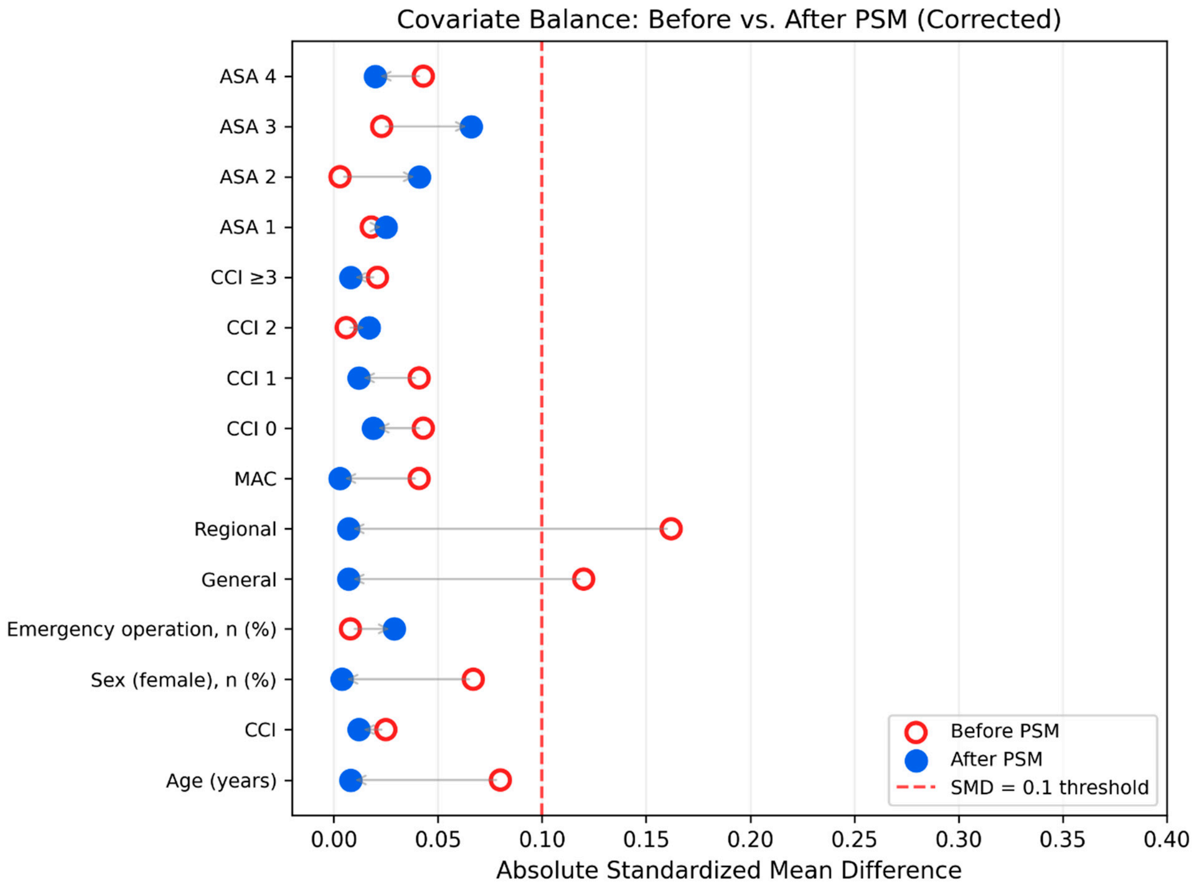

Supplementary Figure S2. Propensity score distributions before and after matching for the crisis and pre-crisis groups.

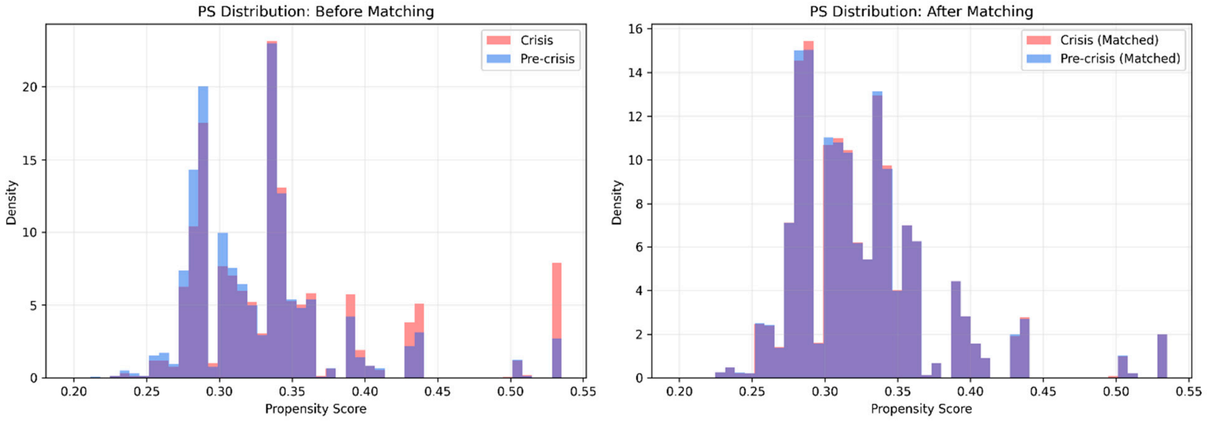

Supplement: Supplementary file 1 [file jcm-15-04261-s001.zip › jcm-4327192-supplementary.pdf]
